# Enhanced Recovery After Surgery (ERAS) protocols following emergency intra-abdominal surgery: A systematic review and meta-analysis protocol

**DOI:** 10.1371/journal.pone.0291140

**Published:** 2023-09-08

**Authors:** Tyler McKechnie, Sameer Parpia, Mohit Bhandari, Joanna C. Dionne, Cagla Eskicioglu

**Affiliations:** 1 Division of General Surgery, Department of Surgery, McMaster University, Hamilton, Ontario, Canada; 2 Department of Health Research Methods, Evidence, and Impact, McMaster University, Hamilton, Ontario, Canada; 3 Division of Orthopedic Surgery, Department of Surgery, McMaster University, Hamilton, Ontario, Canada; 4 Division of General Surgery, Department of Surgery, St. Joseph Healthcare, Hamilton, Ontario, Canada; Queen’s University, CANADA

## Abstract

**Objective:**

The aim of this systematic review and meta-analysis is to evaluate whether the implementation of Enhanced Recovery After Surgery (ERAS) protocols for adult patients undergoing emergency intra-abdominal surgery decreases postoperative length of stay, postoperative morbidity, and mortality compared to conventional perioperative care.

**Methods:**

A systematic review and meta-analysis will be performed and reported in accordance with the Preferred Reporting Items for Systematic Reviews and Meta-Analyses (PRISMA). It has been registered on the International Prospective Register for Systematic Reviews (PROSPERO; CRD42023391709). A comprehensive, electronic search strategy will be used to identify studies published and indexed in MEDLINE, EMBASE, Web of Science, CENTRAL, and Pubmed databases since their inception. Trial registries and references of included studies and pertinent previous systematic reviews will also be searched. Studies will be included if they are randomized controlled trials or cohort studies evaluating adult patients undergoing emergency intra-abdominal surgery and comparing ERAS or modified ERAS protocols to conventional perioperative care and report one of the following outcomes: postoperative length of stay, overall 30-day morbidity, 30-day mortality, 30-day infectious morbidity, prolonged postoperative ileus, return of bowel function, and 30-day readmissions. A meta-analysis will be performed using a random effects model for all comparative data using Cochrane Review Manager 5.3 (London, United Kingdom).

**Discussion:**

ERAS protocols have become standard of care for patients undergoing elective surgery. Their use in the setting of emergency surgery is far less common. The aim of this systematic review and meta-analysis is to assess whether there are benefits in patient important outcomes with the implementation of ERAS protocols for patients undergoing emergency intra-abdominal surgery. Ultimately, we hope to promote their use and further large randomized controlled trials evaluating emergency surgery ERAS programs.

**Prospero registration number:**

CRD42023391709.

## Introduction

In the early 2000s, a collaborative group on perioperative care was formed between surgeons in Denmark, Sweden, and the United Kingdom; this group became known as the Enhanced Recovery After Surgery (ERAS) Study Group [1]. Their mission was to develop multimodal perioperative care pathways for patients undergoing surgery to promote earlier recovery and return to functionality [2]. Their first guidelines were published in 2005 and pertained to patients undergoing elective colorectal surgery [3]. Since this time, they have published numerous guidelines outlining synergistic perioperative interventions aimed at helping patients recover from pancreatectomy, liver transplantation, bariatric surgery, lumbar spinal fusion, and more [4–7].

The various ERAS protocols have become standard of care for patients undergoing elective surgery [8]. Yet, some of the most vulnerable patients that undergo surgery, and those that often fare the worst in terms of postoperative morbidity and recovery, are patients requiring emergent surgery. Perioperative mortality can reach 30% in patients undergoing emergency laparotomy [9]. Originally, it was thought that these patients were too ill to withstand the core principles of ERAS protocols such as early ambulation and enteral feeding [10]. However, studies have begun applying ERAS protocols and modified ERAS protocols to the postoperative care of emergency surgery patients, demonstrating both safety and efficacy [11–14]. Modified ERAS protocols include many of the same components as ERAS protocols, but take into account the limitations in opportunities to provide perioperative care to patients presenting emergently [11]. For example, depending on the patient presentation, many of the preoperative interventions included in ERAS protocols, such as preoperative nutritional supplementation and smoking cessation, cannot be included [15]. The ERAS Study Group has recently published guidelines pertaining to the use of ERAS protocols in patients undergoing emergency laparotomy [16].

Systematic reviews and meta-analyses have published data examining the pooled effect of ERAS protocols on this population [17, 18]. However, there have been recent randomized controlled trials (RCTs) and large observational studies since the most recent data synthesis study [19–22]. Moreover, previous systematic reviews have focused solely on specific diagnoses such as obstructing colorectal cancer or perforated duodenal ulcer. Our aim is to update these previous systematic reviews and meta-analysis and compare adult patients receiving ERAS protocols following all emergency intra-abdominal surgery to those receiving conventional perioperative care. The specific research question is as follows: Does the implementation of ERAS protocols (i.e., a combination of pre-, intra-, and postoperative interventions previously defined by the ERAS Study Group) for adult patients (i.e., 18 years of age and older) undergoing emergency intra-abdominal surgery decrease postoperative length of stay (LOS), postoperative morbidity (both overall postoperative morbidity and system-specific morbidity), and mortality compared to conventional perioperative care (i.e., care at the discretion of the surgeon and/or anesthesiologists, liberal use of lines, tubes, and drains, and lack of early feeding and ambulation)? We hypothesize that perioperative ERAS protocols for emergency intra-abdominal surgery will decrease length of stay, postoperative morbidity, and postoperative mortality in adult patients compared to conventional perioperative care.

## Materials and methods

We will perform a systematic review and meta-analysis comparing ERAS or modified ERAS on post-operative outcomes using a random effects model, and will report out results according to Preferred Reporting Items for Systematic Reviews and Meta-Analyses (PRIMSA) Guidelines. Below we outline our methodology for this review which has been reported according to PRISMA-P (S1 Checklist).

### Intervention

If a study reports the use of an ERAS or modified ERAS protocol that contains preoperative, intraoperative, and postoperative interventions in keeping with those previously defined in ERAS guidelines then it will be considered adequate for inclusion [16, 23]. Given the complex nature of this intervention, we will narratively describe each ERAS protocol from each included study.

### Comparator

Only studies utilizing conventional perioperative care as a comparator group will be considered for inclusion. Conventional perioperative care will vary significantly by institution; thus, an exact definition is not possible. If conventional perioperative care is described as significantly different across preoperative, intraoperative, and postoperative phases of care by the study authors, it will be considered adequate for the purposes of this review. We will narratively describe each conventional perioperative care protocol from each included study.

### Search strategy

The search was designed and conducted by a medical research librarian with input from study investigators. Search terms included “general surgery”, “enhanced recovery after surgery”, “ERAS”, and more (complete search strategies for each of the individual database searches are available in Tables 1–4).

**Table 1:** Complete search strategy (Medline and Embase database example).

1. General Surgery/
2. Colorectal Surgery/
3. Traumatology/
4. Surgical Oncology/
5. Thoracic Surgery/
6. Digestive System Surgical Procedures/
7. Laparoscopy/
8. Laparotomy/
9. Hand-Assisted Laparoscopy/
10. Trauma Surg*.mp.
11. Hepatobiliary Surg*.mp.
12. Bowel Resect*.mp.
13. Emergencies/
14. Emerg*.mp.
15. Emergency Surg*.mp.
16. Urgent.mp.
17. Urgent Surg*.mp.
18. Acute Care Surg*.mp.
19. Enhanced Recovery After Surgery/
20. ERAS.mp.
21. Enhanced Recovery Protocol*.mp.
22. Enhanced Recovery Pathway*.mp.
23. ERP.mp.
24. Enhanced Recovery.mp.
25. Fast-track Recovery.mp.
26. Or/1-12
27. Or/13-18
28. Or/19-25
29. 26 and 27 and 28
30. Animals/
31. Humans/
32. 30 not (30 and 31)
33. 29 not 32

**Table 2:** Complete search strategy (CENTRAL database example).

(“emergency general surgery”):ti,ab,kw OR (“emergency bowel resection”):ti,ab,kw OR (“acute care surgery”):ti,ab,kw (word variations have been searched)
AND
(“enhanced recovery after surgery”):ti,ab,kw OR (“enhanced recovery protocols”):ti,ab,kw (word variations have been searched)

**Table 3:** Complete search strategy (Pubmed database example).

(“General Surgery”) OR (Bowel Resect*) OR (“Colorectal Surgery”) OR (“Trauma Surgery”) OR (“Surgical Oncology”) OR (“Hepatobiliary Surgery”) OR (“Thoracic Surgery”) OR (“Gastrointestinal Surgery”) OR (Laparoscop*) OR (Laparotom*)
AND
(Emerg*) OR (“Emergency Surgery”) OR (Urgen*) OR (“Urgent Surgery”) OR (“Acute Care Surgery”)
AND
(“Enhanced Recovery After Surgery”) OR (“ERAS”) OR (“Enhanced Recovery Pathways”) OR (“Enhanced Recovery Protocols”) OR (“ERP)

**Table 4:** Complete search strategy (Web of Science database example).

TS = (“General Surgery”) OR TS = (“Colorectal Surgery”) OR TS = (“Thoracic Surgery”) OR TS = (“Surgical Oncology”) OR TS = (“Trauma Surgery”) OR TS = (“Hepatobiliary Surgery”) OR TS = (bowel resect*) OR TS = (Laparoscop*) OR TS+(Laparotom*)
AND
TS = (Emergency) OR TS = (Urgency) OR TS = (“Emergency Surgery”) OR TS = (“Urgent Surgery”) OR TS = (“Acute Care Surgery”)
AND
TS = (“Enhanced Recovery”) OR TS = (“Enhanced Recovery After Surgery”) OR TS = (“ERAS”) OR TS = (“ERP”) OR TS = (“Enhanced Recovery Pathway”) OR TS = (“Enhanced Recovery Protocol”) OR TS = (“Fast-Track”)

### Information sources

The following databases will be searched from inception:

1. Medline
2. Embase
3. Cochrane Central Register of Controlled Trials (CENTRAL)
4. Web of Science
5. Pubmed

The following trial registries will be searched from inception:

1. Clinicaltrials.gov
2. International Clinical Trials Registry Platform

The references of studies meeting inclusion criteria as well as previous pertinent systematic reviews were searched manually to ensure that all relevant articles were included.

### Study selection

Two reviewers will independently evaluate the systematically searched titles and abstracts using a standardized, pilot-tested form. Discrepancies that occur at the title and abstract screening phases will be resolved by inclusion of the study. At the full-text screening stage, discrepancies will be resolved by consensus between the reviewers. If disagreement persists, an additional reviewer will be consulted.

### Eligibility criteria

The inclusion criteria will be as follows:

1. RCTs, prospective cohort studies, or retrospective cohort studies.
2. Studies evaluating adult patients (i.e., 18 years of age and older) undergoing emergency intra-abdominal surgery.
3. Studies comparing ERAS or modified ERAS protocols and conventional postoperative care.
4. Studies reporting any of the defined outcomes for this systematic review (see below).

The exclusion criteria will be as follows:

1. Systematic reviews, meta-analysis, case-control, case series, case study, surveys, letters, editorials, conference abstracts, or any other type of study not reporting primary data.
2. Single-armed, non-comparative studies.
3. Studies not evaluating patients undergoing emergency intra-abdominal surgery.
4. Studies evaluating ERAS protocols following elective surgery.
5. Studies including pediatric patients (i.e., 18 years of age or younger).
6. Studies not reporting of any of the defined outcomes for this systematic review (see below).

### Data management and items

Two reviewers will independently conduct data extraction into a data collection form designed *a priori*. Discrepancies will be reviewed in detailed and discussed until consensus is reached. The extracted data will include:

1. Study characteristics: author, year of publication, study period, study design, location of study, number of included centers, study inclusion criteria, study exclusion criteria, primary outcomes, secondary outcomes, statistical analyses.
2. Patient demographics: age, sex, body mass index [BMI], comorbidities, Charlson Comorbidity Index, American Society of Anesthesiologist Score, preoperative blood work.
3. Disease characteristics: type of disease, stage of malignancy presenting symptoms.
4. Surgical characteristics: type of surgery, operative approach.
5. ERAS program details: pre-, intra-, and postoperative interventions included in ERAS protocols.
6. Comparator program details: pre-, intra-, and postoperative interventions included in comparator protocols.
7. Postoperative outcomes: 30-day mortality, overall 30-day morbidity, Clavien-Dindo morbidity grades, 30-day infectious morbidity, 30-day system specific morbidity, return of bowel function, postoperative LOS, 30-day readmission rate, 30-day reoperation rate, discharge disposition.

### Outcomes

The following outcomes will be extracted and analyzed in this systematic review and meta-analysis:

1. Postoperative LOS will be defined as the number of days from the index procedure to the time the patient leaves an acute care bed.
2. Overall postoperative morbidity will include any deviation from the expected postoperative course as reported by each included study. In the majority of included studies, postoperative morbidity was a composite of system-specific complications, including cardiovascular, respiratory, gastrointestinal, genitourinary, infectious, and wound-complications. If this was not reported as a pooled outcome in the included study (i.e., overall postoperative morbidity), then the outcome was recorded as missing.
3. Postoperative mortality will include any death reported by each included study.
4. Postoperative infectious morbidity will be defined as any deviation from the expected postoperative course due to infectious complication as reported by each included study. If a study does not report overall postoperative infectious morbidity, then the following order of priority for infectious outcomes will be utilized to represent the event rate: i) overall surgical site infection (SSI), ii) organ space SSI, iii) deep SSI, iv) anastomotic/repair leak, v) superficial SSI, vi) pulmonary infection, vii) urinary tract infection.
5. PPOI was defined as at least two of the following occurring on or after postoperative day four: (1) abdominal distention; (2) inability to tolerate oral diet; (3) nausea or vomiting; (4) insertion of a nasogastric tube; (5) radiographic examination in keeping with PPOI [24]. If studies do not report incidence of PPOI, we will use reinsertion of nasogastric tube as the outcome to estimate effect.
6. Time to return of bowel function will be defined as number of days to passage of flatus. If studies did not report number of days to passage of flatus, we will use number of days to tolerance of solid oral intake as the outcome to estimate effect.
7. 30-day readmission will be defined as readmission to hospital reported by each included study within 30 days of the index surgery.

### Risk of bias assessment

Risk of bias for RCTs will be assessed using the Cochrane Risk of Bias Tool for Randomized Controlled Trials 2.0 [25]. Two reviewers will assess the risk of bias independently. Discrepancies will be discussed amongst the reviewers until consensus is reached. Risk of bias figures will be created with RoBvis [26].

### Data synthesis

All statistical analyses will be performed on STATA version 15 (StataCorp, College, TX) and Cochrane Review Manager 5.3 (London, United Kingdom). The threshold for statistical significance will be set *a priori* at a p of <0.05. A pairwise meta-analysis will be performed using an inverse variance random effects model for all comparative data. Pooled effect estimates will be obtained by calculating the risk ration (RR) along with their respective 95% confidence intervals (CI) to confirm the effect size estimation. Mean and standard deviation (SD) will be estimated for studies that only report median and interquartile range (IQR) using the method described by Wan *et al*. [27]. Missing SD data will be calculated according to the prognostic method [28]. Assessment of heterogeneity will be completed using the inconsistency (I^2^) statistic. An I^2^ greater than 40% will be considered to represent considerable heterogeneity [29]. Bias in meta-analyzed outcomes will be assessed with funnel plots when data from more than 10 studies are included in the meta-analysis [30].

The following sub-group analyses are planned to explore heterogeneity:

1. Type of disease (i.e., upper gastrointestinal, lower gastrointestinal, general surgery, trauma)
2. Operative approach (i.e., more than 50% laparoscopic vs. less than 50% laparoscopic)
3. Age (i.e., mean age greater than 60 vs. mean age less than 60)
4. Geographic location of study (i.e., Asia, North America, Other)

The following sensitivity analyses are planned:

1. Leave-one-out

### Certainty of evidence

Certainty of evidence for estimates derived from meta-analyses will be assessed by Grading of Recommendations, Assessment, Development and Evaluation (GRADE) [31]. The certainty of the evidence will be scored as high, moderate, low, or very low for each outcome according to six pre-specified categories (i.e., risk of bias, inconsistency of results, directness of evidence, imprecision, publication bias, and other). The GRADE results will be collated in a summary of findings table, as recommended by the Cochrane Collaborative. The calculations and organization of results into a summary of findings table will be done using the GRADEPro software [32].

## Discussion

ERAS programs have become standard of care across numerous elective surgeries. While the use of these programs in the emergent setting is far less frequent, there are some existing data investigating their use in patients undergoing emergency intra-abdominal surgery. Our aim is this systematic review and meta-analysis will amalgamate previously published data to highlight the potential benefits associated with the implementation of ERAS programs for patients undergoing emergency surgery. Ultimately, highlighting the potential benefit of these programs in the perioperative care of emergency surgery patients, will promote their use, identify knowledge gaps, and contribute to further study by way of large randomized controlled trials to address these gaps to further establish ERAS programs for these patients around the world.

## Supporting information

S1 Checklist PRISMA-P (Preferred Reporting Items for Systematic review and Meta-Analysis Protocols) 2015 checklist: Recommended items to address in a systematic review protocol*. (DOC)
